# Functional exosome-mediated co-delivery of doxorubicin and hydrophobically modified microRNA 159 for triple-negative breast cancer therapy

**DOI:** 10.1186/s12951-019-0526-7

**Published:** 2019-09-03

**Authors:** Chunai Gong, Jing Tian, Zhuo Wang, Yuan Gao, Xin Wu, Xueying Ding, Lei Qiang, Guorui Li, Zhimin Han, Yongfang Yuan, Shen Gao

**Affiliations:** 1 Department of Pharmaceutics, Changhai Hospital, Second Military Medical University, Shanghai, 200433 People’s Republic of China; 2 Department of Pharmacy, Shanghai Ninth People’s Hospital, Shanghai Jiao Tong University School of Medicine, Shanghai, 200011 People’s Republic of China; 3 Department of Clinical Pharmacy and Pharmaceutical Management, School of Pharmacy, Fudan University, Shanghai, 201203 People’s Republic of China; 4 Department of Clinical Pharmacy, Shanghai General Hospital, School of Medicine, Shanghai Jiao Tong University, Shanghai, 200080 People’s Republic of China

**Keywords:** Exosome, MicroRNA 159, Doxorubicin, Triple-negative breast cancer, Co-delivery

## Abstract

Exosomes (Exo) hold great promise as endogenous nanocarriers that can deliver biological information between cells. However, Exo are limited in terms of their abilities to target specific recipient cell types. We developed a strategy to isolate Exo exhibiting increased binding to integrin α_v_β_3_. Binding occurred through a modified version of a disintegrin and metalloproteinase 15 (A15) expressed on exosomal membranes (A15-Exo), which facilitated co-delivery of therapeutic quantities of doxorubicin (Dox) and cholesterol-modified miRNA 159 (Cho-miR159) to triple-negative breast cancer (TNBC) cells, both in vitro and in vivo. The targeted A15-Exo were derived from continuous protein kinase C activation in monocyte-derived macrophages. These cell-derived Exo displayed targeting properties and had a 2.97-fold higher production yield. In vitro, A15-Exo co-loaded with Dox and Cho-miR159 induced synergistic therapeutic effects in MDA-MB-231 cells. In vivo, miR159 and Dox delivery in a vesicular system effectively silenced the TCF-7 gene and exhibited improved anticancer effects, without adverse effects. Therefore, our data demonstrate the synergistic efficacy of co-delivering miR159 and Dox by targeted Exo for TNBC therapy.

## Background

Triple-negative breast cancer (TNBC) comprises approximately 15% of all breast cancer (BC) cases and shows a higher morbidity because of its aggressive behavior, poor prognosis, and lack of targeted treatments [1]. TNBC is immunohistochemically defined by the lack of human epidermal growth factor receptor 2, estrogen receptor, and progesterone receptor expression [2]. The only modalities of systemic therapy available for TNBC are chemotherapy with platinum [3, 4] or untargeted chemotherapy (alone or in combination), providing limited choices with evident side effects [5, 6]. Hence, a safe and efficient targeted delivery system is critically needed for TNBC therapy.

Exosomes (Exo) are membrane-derived vesicles, ~ 40–200 nm in diameter, that are secreted by many cell types and are present in vitro and in vivo [7, 8]. Exo are thought to contribute to homeostasis and disease development (including cancer and neurodegenerative disorders) [9–11]. Thus, Exo may serve as a endogenous vectors in delivering therapeutic drugs such as chemotherapeutic agents, anti-inflammatory drugs, short-interfering RNAs (siRNAs), and microRNAs (miRNAs) [12–16]. To confer targeting capability to Exo, a disintegrin and metalloproteinase 15 (A15) was previously expressed in human macrophage-derived Exo (A15-Exo). A15, the only ADAM protein containing an Arg-Gly-Asp (RGD) motif in its disintegrin-like domain, is a widely expressed membrane protein that is involved in tumor progression and suppression [17–19]. Exo rich in A15 displayed an enhanced binding affinity for integrin α_v_β_3_ in an RGD-dependent manner [20, 21]. The RGD served as the targeting moiety to enhance the cellular uptake of Exo by integrin α_v_β_3_-overexpressing tumor, including melanoma, glioma, and BC [22–24].

MiRNA dysregulation has been implicated in tumor initiation, progression, and metastasis in several cancer types, including BC [25–30]. Cholesterol-modified RNAs are asymmetric oligonucleotides with hydrophobic-moiety modifications that improve their stability, promote cellular internalization [31–34] and target gene silencing [35, 36]. Wang et al. [37] found that the levels of miRNA 159 (miR159) inversely correlated with BC incidence and progression. Furthermore, transcription factor 7 (TCF7) were identified as putative miR159 target, that is a TCF/LEF-family transcription factor of the Wnt-signaling pathway, and was identified as one of several overexpressed Wnt-pathway genes in TNBC [38–42].

**Scheme 1:**
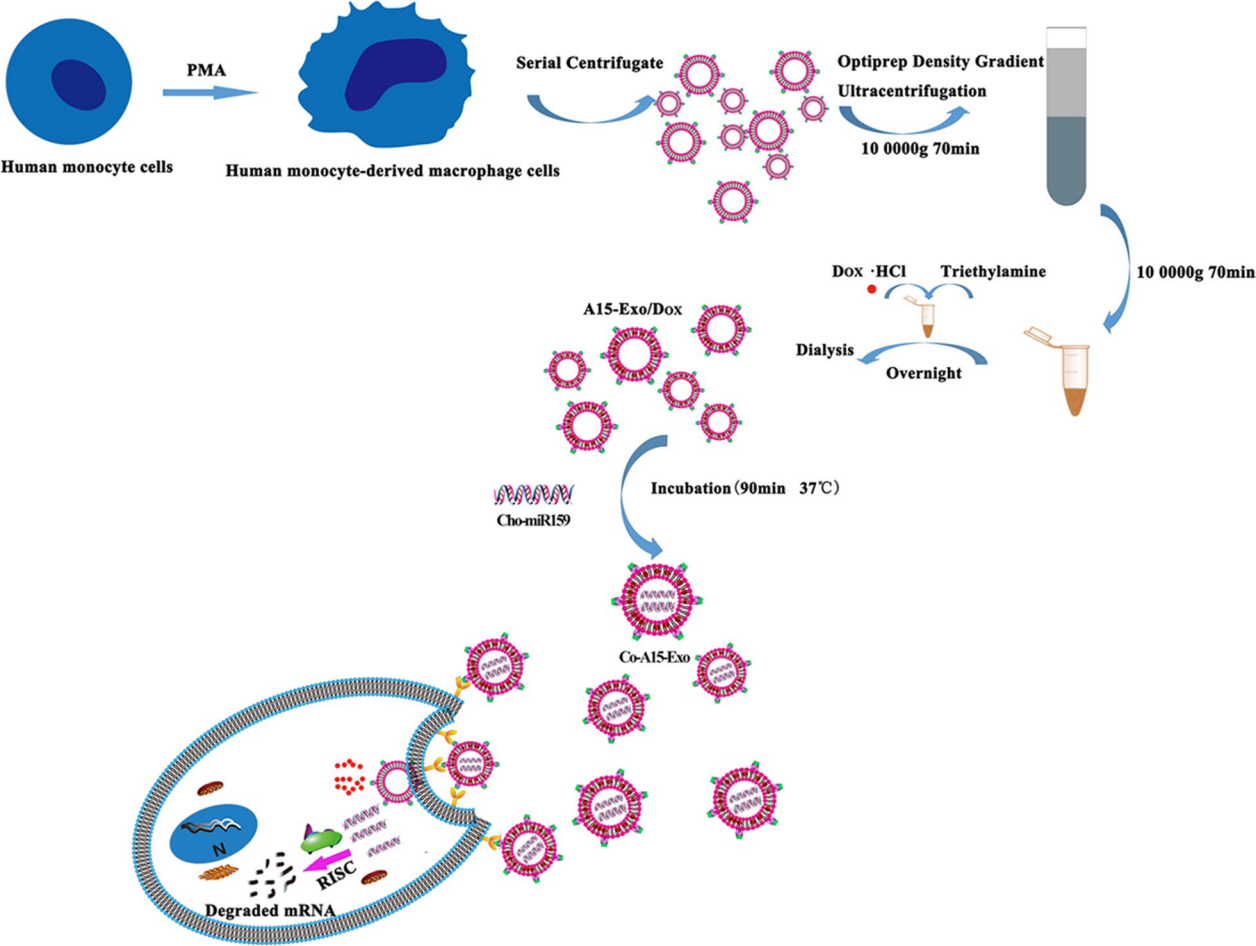
Formation and release of Dox and Cho-miR159-loaded A15-Exo (Co-A15-Exo)

Herein, we developed a strategy to produce nanoscale target-specific Exo to co-deliver cholesterol-modified miRNA and chemotherapeutic drugs to TNBC cells. Our previous work showed that the therapeutic strategy of co-delivering genes and chemotherapeutic drugs can produce synergistic effects against cancer [43, 44]. Target-specific Exo were generated at a high yield by stimulating THP-1 cells with phorbol 12-myristate 13-acetate (PMA), which effectively increased exosomal A15 release. Doxorubicin hydrochloride (Dox) packaging into A15-Exo was achieved by mixing an appropriate concentration of Dox with Exo in a triethylamine solution overnight. Then, we co-incubated cholesterol-modified mi159 (Cho-miR159) with A15-Exo/Dox to form a co-delivery system (Co-A15-Exo, Scheme 1). These extracellular vesicles were evaluated in terms of their targeting and therapeutic effects, both in vitro and in vivo.

## Results

### PMA-induced monocyte differentiation

Previously, PMA treatment resulted in a differentiated phenotype, which was determined by evaluating the expression of the surface marker CD11b [45]. Kim et al. [19] reported that stimulation of THP-1 cells with PMA effectively increased exosomal A15 release during the process of differentiation. We found that PMA exposure significantly increased CD11b expression in THP-1 cells in a concentration-dependent manner, whereas A15 expression was slightly affected by PMA treatment (Additional file 1: Figure S1A). However, when PMA-differentiated (PMA: 50 ng/mL) THP-1 cells were washed with phosphate-buffered saline (PBS) and further cultured for 1–5 days, A15 expression increased significantly during culture days 2–3, after which A15 expression peaked for the next 2 days (Additional file 1: Figure S1Ba, S1Bb).

### Characterization of Exo complexes

**Fig. 1:**
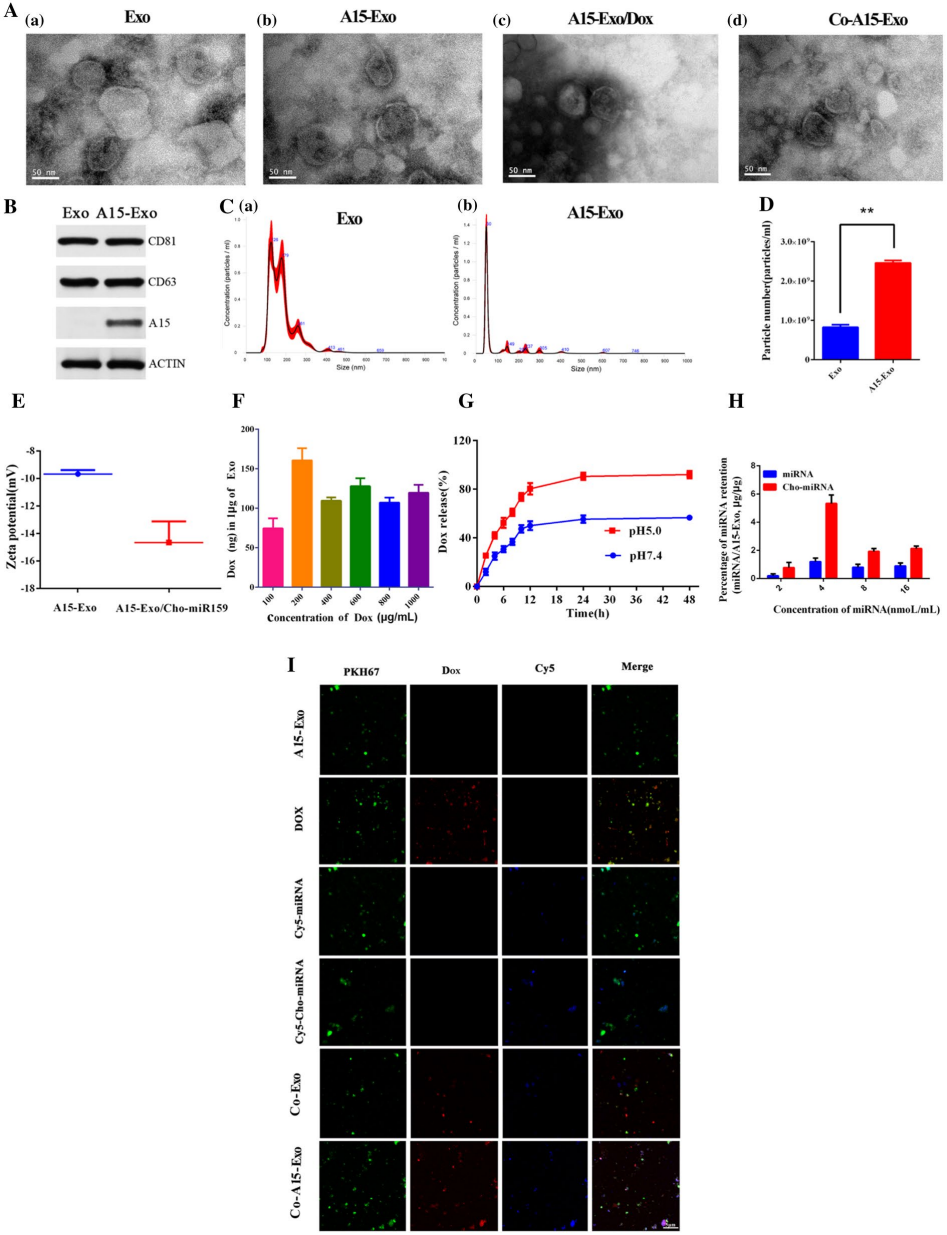
Characterization of Dox and Cho-miR159 loaded A15-Exo. **A** Representative TEM images of Exo (a), A15-Exo (b), A15-Exo/Dox (c), and Co-A15-Exo (d). **B** Western blot analysis of Exo- and A15-Exo-marker proteins CD81, CD63, and A15. **C** Size distributions of Exo (a) and A15-Exo (b) measured by NTA. **D** The numbers of Exo and A15-Exo produced from the same number (1 × 10^7^) of THP-1 monocytes or differentiated macrophages (treated with 50 ng/mL PMA). **E** Zeta potentials of A15-Exo and A15-Exo/Cho-miR159. **F** Quantification of Dox packaged into A15-Exo when incubated with 100, 200, 400, 600, 800, or 1000 μg/mL of Dox. **G** Dox-release profiles of A15-Exo after incubation for up to 48 h in PBS at pH 5.0 or pH 7.4 at 37 °C (n = 3). Data are presented as the mean ± SD. **H** Optimized parameters for introducing miRNA or Cho-miRNA into A15-Exo by co-incubation. **I** Representative fluorescence microscopy images of Exo/Cy5-Cho-miRNA, A15-Exo/Cy5-Cho-miRNA, Co-Exo, and Co-A15-Exo. PKH67, Cy5, and Dox are shown as green, blue, and red fluorescent signals, respectively. The merged signals for PKH67 and Dox are indicated by yellow fluorescence. White fluorescence resulted from the overlay of red, green, and blue fluorescence in the merged image. Scale bars: 5 μm. *Exo* exosomes, *A15* A disintegrin and metalloproteinase 15, *Dox* doxorubicin

We purified Exo and A15-Exo from the culture supernatants of untreated or differentiated THP-1 cells by ultracentrifugation. Transmission electron microscopy (TEM) observations showed that Exo (Fig. 1Aa), A15-Exo (Fig. 1Ab), A15-Exo/Dox (Fig. 1Ac), and Co-A15-Exo (Fig. 1Ad) displayed characteristic saucer-like bilayer membrane structures, which demonstrates that Exo remained intact when loaded with Dox or Cho-miRNA. As shown in Fig. 1B, the purified Exo contained major exosomal marker proteins, including CD81 and CD63. Released A15 was detected along with CD81 and CD63, indicating that released A15 was an exosomal component. In addition, nanoparticle-tracking analysis (NTA) showed that the Exo exhibited narrow size distribution, with mean particle diameters of 179.4 ± 65.5 nm and 94.1 ± 104.4 nm for Exo and A15-Exo, respectively; further, there were no significant differences between size distribution in the exosome types (*p* > 0.05) (Fig. 1Ca, b). In terms of Exo numbers, 2.46 × 10^9^ A15-Exo were produced, while only 8.27 × 10^8^ Exo were produced from the same number (1 × 10^7^) of THP-1 monocytes or differentiated macrophages (treated with 50 ng/mL PMA), respectively, as shown in Fig. 1D.

Figure 1E shows that the zeta potential decreased from − 9.68 ± 0.29 mV (with A15-Exo) to − 14.67 ± 1.53 mV (with A15-Exo/Cho-miR159). This reduction in the zeta potential for A15-Exo/Cho-miR159 may have resulted from the presence of negatively charged Cho-miR159, similar to previous findings [46].

### Drug loading and release

Dox loading into A15-Exo was dependent on the concentration used for incubation. For example, 74.5 ± 12.9 ng, 160.6 ± 15.4 ng, 109.5 ± 4.2 ng, 127.9 ± 9.9 ng, 107.2 ± 6.4 ng, or 119.6 ± 10.0 ng of Dox was loaded into 1 μg of A15-Exo (measured based on the total protein concentration) when 100, 200, 400, 600, 800, or 1000 μg/mL of Dox was used, respectively (Fig. 1F). In this study, we used A15-Exo/Dox prepared by incubation with 200 μg/mL of Dox, which showed maximal loading of ~ 160 ng Dox in 1 μg Exo.

The Dox-release profiles of Co-A15-Exo were investigated at pH 7.4 (physiological environment) and at pH 5.0 (late endosomal and lysosomal environments) at 37 °C [47]. As shown in Fig. 1G, Dox release from Co-A15-Exo reached 90.5% at pH 5.0, but only 55.3% at pH 7.4 (*p* < 0.01) after 24 h. The pH-dependent behavior of Dox release agreed with our previous study [48, 49].

Hydrophobically modified miRNAs are asymmetric miRNAs, where the 3′ end of the passenger strand is conjugated to a bioactive hydrophobic cholesterol molecule. The cholesterol group could enable quick membrane association and facilitate internalization [31, 50]. As shown in Fig. 1H, the miR159-loading efficiency into A15-Exo was closely related to the miRNA concentration. For example, the miR159-loading efficiency into A15-Exo was approximately 1.2%, whereas the loading efficiency of Cho-miR159 was approximately 5.33%. We measured co-localization of PKH67 (an exosomal marker) and Cy5-Cho-miRNA or Dox to evaluate the impact of exosomal loading on the kinetics. White fluorescence (Co-A15-Exo-treated group), resulting from the overlay of red, blue, and green fluorescence in the merged image, indicated that Dox and Cy5-Cho-miRNA were effectively co-delivered into Exo (Fig. 1I). Cyan fluorescence (overlay of blue and green fluorescence) in the Cy5-Cho-miRNA-treated group was stronger than that of the Cy5-miRNA group after a 90-min incubation. These data indicated that the cholesterol moiety facilitated Cy5-miRNA internalization into A15-Exo.

### In vitro targeting of A15-Exo

The distribution characteristics of A15-Exo and Exo were analyzed after 4-h incubations with MDA-MB-231 and MCF-7 cells. As shown in Additional file 1: Figure S2, A15-Exo displayed higher internalization in MDA-MB-231 cells. Moreover, compared to Exo, more pronounced cell-surface fluorescence was observed in the A15-Exo group, indicating the enhanced affinity of A15-Exo with MDA-MB-231 cells. For MCF-7 cells with little α_v_β_3_ expression, no difference was found between A15-Exo and Exo in confocal microscopy images.

**Fig. 2:**
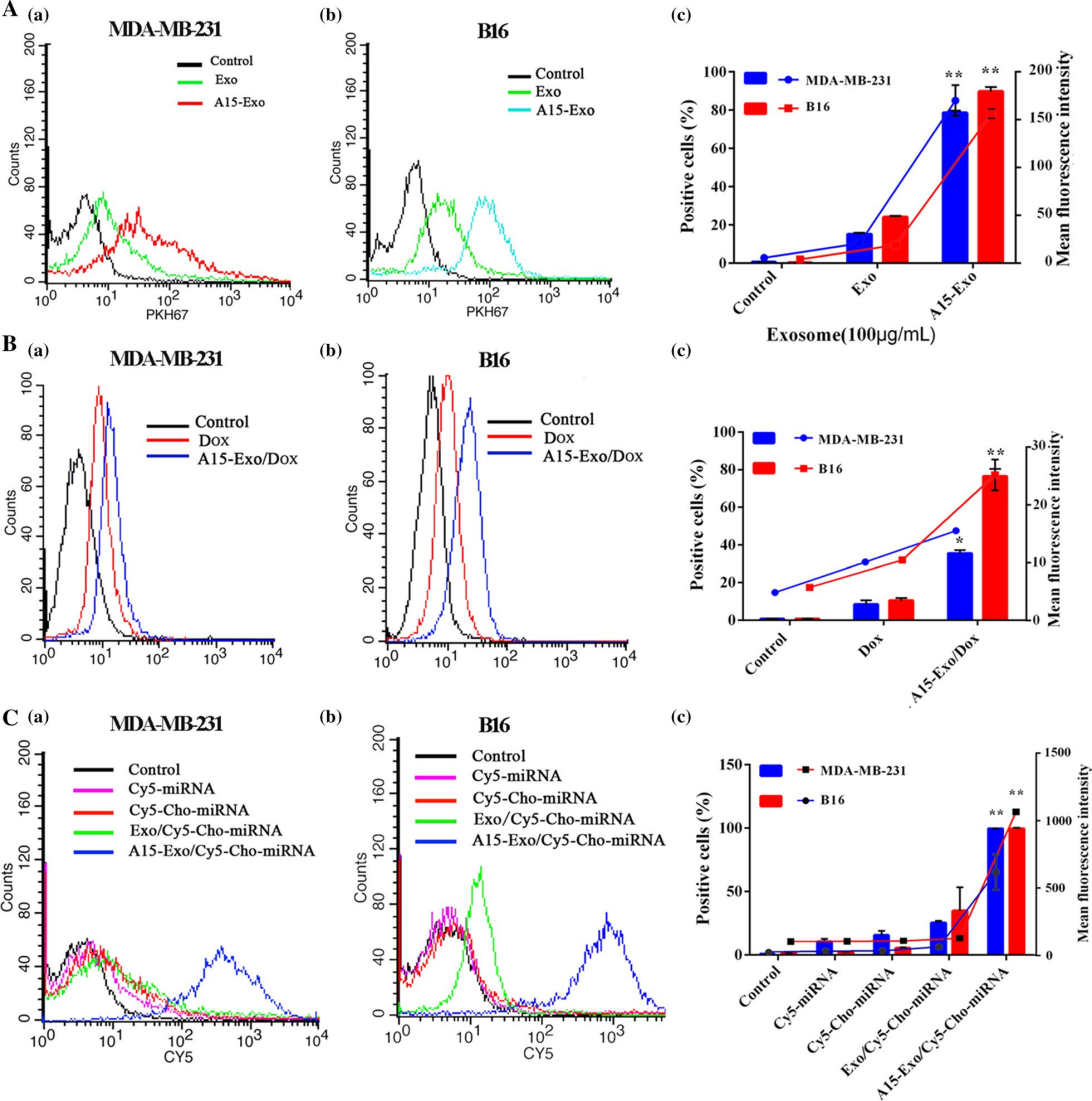
Internalization of Dox and Cy5-Cho-miR159 mediated by Exo or A15-Exo. **A** Intracellular uptake of PKH67-labeled Exo or A15-Exo by MDA-MB-231 and B16 cells, as determined by flow cytometry. (a, b) Flow cytometry data. (c) Quantitative analysis of the percentage of PKH67-positive cells and mean fluorescence intensity. **B** Cellular uptake of Dox mediated by Exo or A15-Exo. Quantitative analysis of the mean fluorescence intensity of Dox in (a) MDA-MB-231 and (b) B16 cells. (c) Quantitative analysis of the percentage of Dox-positive cells. **C** Cellular uptake of Cy5-miRNA or Cy5-Cho-miRNA mediated by Exo or A15-Exo. Quantitative analysis of the mean fluorescence intensities of Cy5-miRNA or Cy5-Cho-miR159 in MDA-MB-231 (a) and B16 (b). (c) Quantitative analysis of the percentages of Cy5-miRNA- and Cy5-Cho-miR159-positive cells

To investigate the cellular uptake of Exo and A15-Exo, Exo and A15-Exo were labeled with PKH67. Exo and A15-Exo were incubated with MDA-MB-231 and B16 cells for 4 h. The flow cytometry data shown in Fig. 2A indicate that a high level of uptake occurred for A15-Exo (approximately 78.60 ± 1.15% in MDA-MB-231 cells and 89.76 ± 2.25% in B16 cells). However, the flow cytometry demonstrated that A15-Exo bound to MDA-MB-231 cells and B16 cells more efficiently than Exo (78.60% vs. 15.23% in MDA-MB-231 cells and 89.76% vs. 24.13% in B16 cells) (Fig. 2Ac), indicating that employing A15 as the ligand for integrin α_v_β_3_ dramatically enhanced the binding ability of Exo to target cells.

### Cellular uptake of Cho-miRNA and Dox

To investigate the cellular uptake of Dox and Cho-miRNA mediated by Exo, Cho-miRNA was first labeled with a Cy5 probe. A15-Exo/Dox, Dox, Cy5-miRNA, Cy5-Cho-miRNA, Exo/Cy5-Cho-miRNA, and A15-Exo/Cy5-Cho-miRNA were incubated with MDA-MB-231 cells and B16 cells for 4 h. The flow cytometry data shown in Fig. 2B, C indicated that A15-Exo/Dox and A15-Exo/Cy5-Cho-miRNA were efficiently internalized (approximately 35.48 ± 1.72% and 99.55 ± 0.18% in MDA-MB-231 cells, respectively, and 76.43 ± 3.92% and 99.53 ± 0.67% in B16 cells, respectively). These data suggest that Dox and Cho-miRNA could be taken up by cells efficiently when delivered via A15-Exo (*p* < 0.01).

### Confocal microscopy

**Fig. 3:**
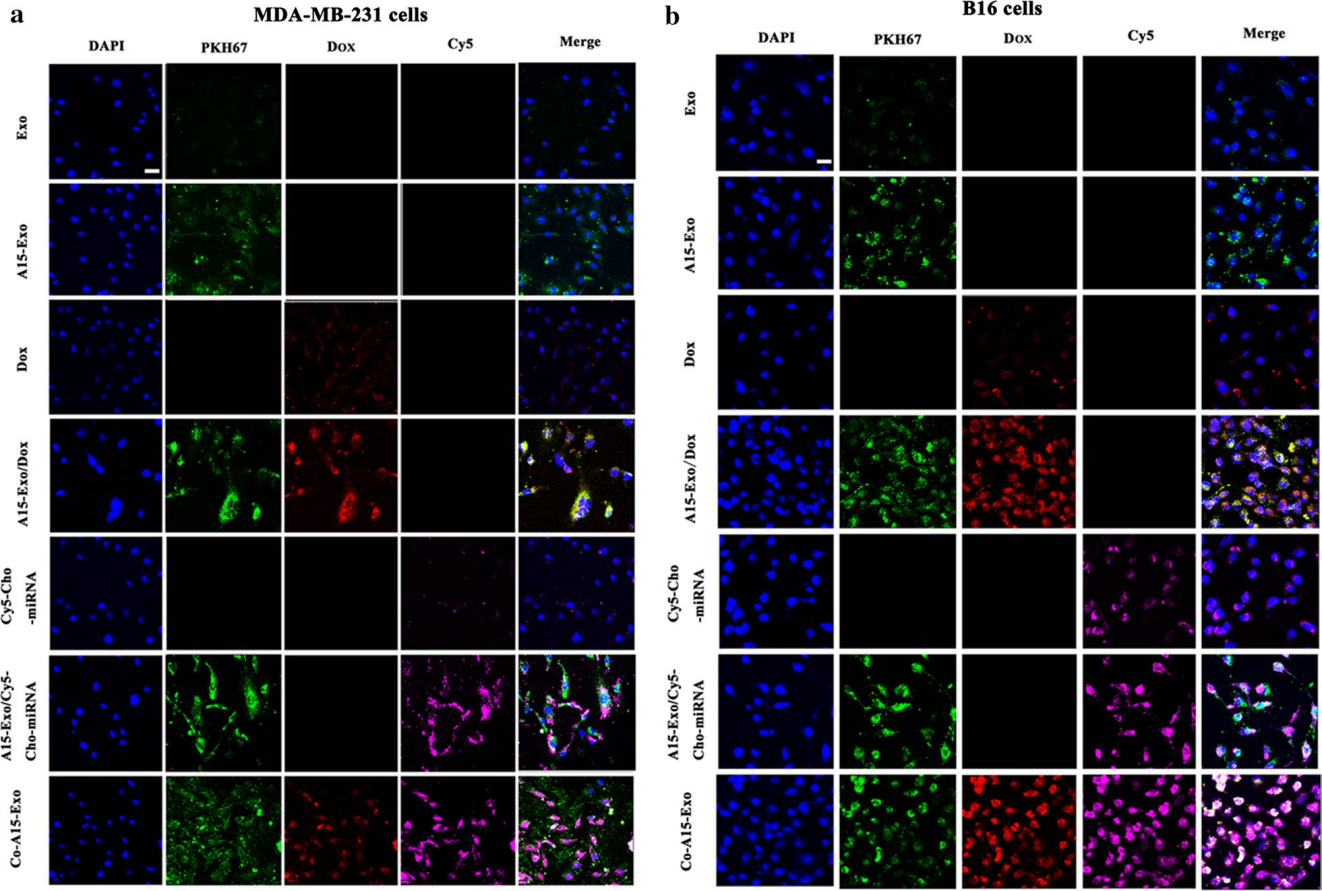
Confocal microscopy images about the intracellular distribution PKH67-labeled drug-loaded Exo or A15-Exo. Images of MDA-MB-231 cells (**a**) and B16 cells (**b**) after incubation with Exo, A15-Exo, Dox, A15-Exo/Dox, Cy5-Cho-miRNA, A15-Exo/Cy5-Cho-miRNA, or Co-A15-Exo for 4 h. Green fluorescence represents PKH67-labeled Exo, red fluorescence represents Dox, and blue fluorescence represents the cell nucleus. Purple fluorescence represents Cy5-labeled miRNA or Cho-miRNA. Scale bar, 20 μm

Confocal laser-scanning microscopy was performed to analyze the subcellular-distribution characteristics of Dox and Cho-miRNA delivered by A15-Exo. The nuclei were stained blue by 4′,6-diamidino-2-phenylindole, the red fluorescence corresponded to Dox, the purple fluorescence corresponded to Cy5, and the green fluorescence corresponded to Exo or A15-Exo (Fig. 3a, b). After a 4-h incubation with Co-A15-Exo, red fluorescence (Dox), green fluorescence (PKH67-labeled A15-Exo), and purple fluorescence (Cy5-Cho-miRNA) were observed in the nuclear and perinuclear regions of the cytoplasm. The white fluorescence, resulting from the overlay of red, blue, purple, and green fluorescence in the merged image, indicated that Dox and Cy5-Cho-miRNA were effectively co-delivered into MDA-MB-231 and B16 cells.

### Cell-viability assay

**Fig. 4:**
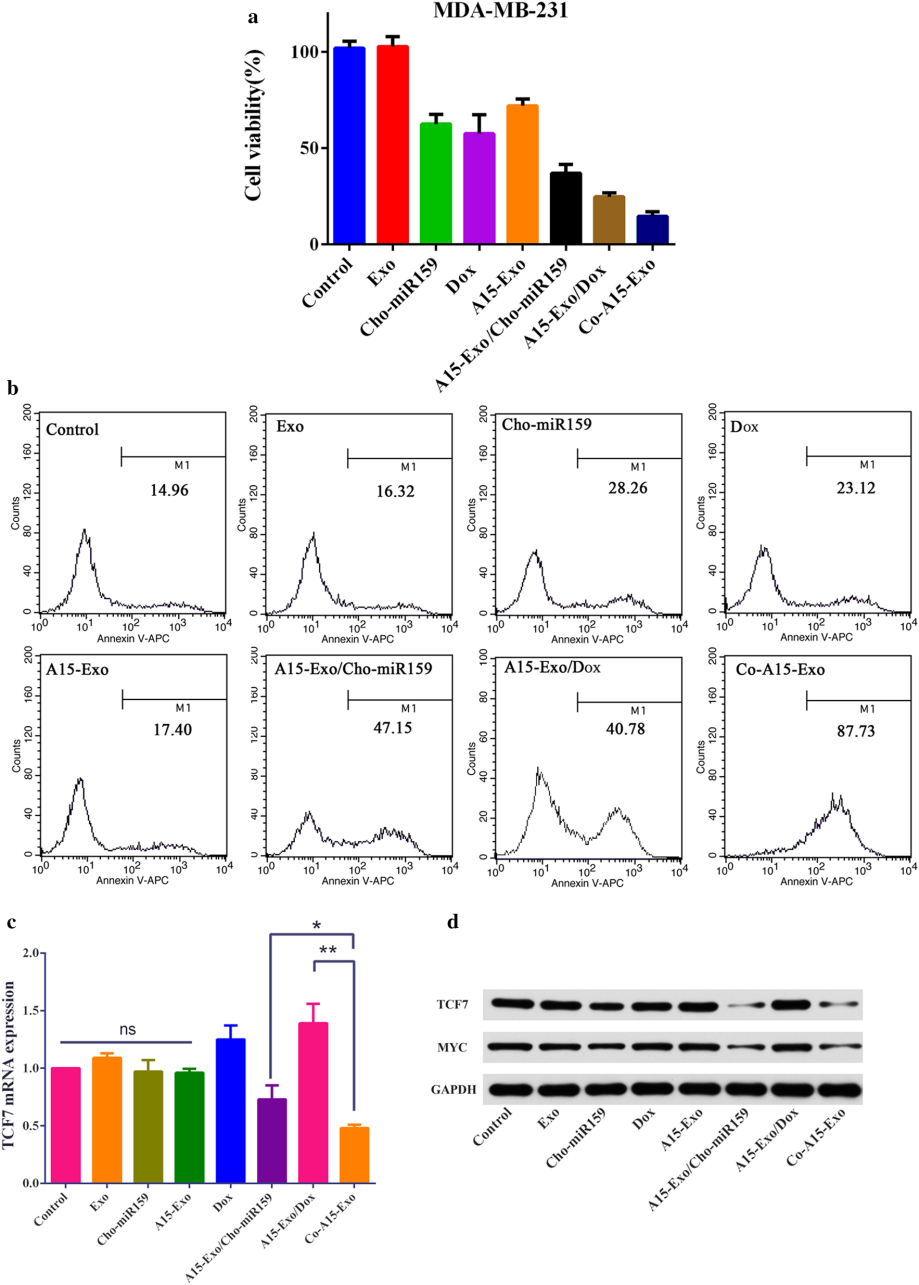
Antitumor efficacy of Co-A15-Exo in vitro. **a** In vitro viability of MDA-MB-231 cells incubated with control medium (control), Exo, Cho-miR159, Dox, A15-Exo, A15-Exo/Cho-miR159, A15-Exo/Dox, or Co-A15-Exo (2 mM Dox and 500 fmoL/mL Cho-miR159). Cell viability was assessed by performing CCK-8 assays. **b** Analysis of apoptosis in MDA-MB-231 cells treated by different formulations for 48 h, as determined by flow cytometry after staining for annexin V expression and PI staining. **c** Relative expression levels of TCF7 mRNA were measured by quantitative real-time PCR after treatment with different formulations in MDA-MB-231 cells. Data are expressed as the mean ± SD (n = 3). **d** TCF7 and MYC expression were evaluated by western blot analysis

We then analyzed the ability of Co-A15-Exo to inhibit cancer cell proliferation. MDA-MB-231 cells were treated with control medium, Exo, Cho-miR159, Dox, A15-Exo, A15-Exo/Cho-miR159, A15-Exo/Dox, or Co-A15-Exo for 48 h, and cell viabilities were measured by performing CCK-8 assays. Co-A15-Exo inhibited cell proliferation with an efficiency comparable to that of free Dox (Fig. 4a), whereas no significant inhibition of cell growth was observed with the Exo- or A15-Exo-treated groups. These data indicated that little or no toxicity was associated with the Exo themselves and that the A15-Exo used in this study had no effect on MDA-MB-231 cell proliferation.

### Apoptosis assay

To evaluate the effects of Dox and Cho-miR159-loaded A15-Exo on MDA-MB-231 cell apoptosis, apoptosis was monitored by flow cytometry after staining cells with an allophycocyanin (APC)-conjugated anti-annexin V antibody and propidium iodide (PI). As shown in Fig. 4c, the control and Exo groups showed a negligible amount of apoptotic and necrotic cells, which indicated that unloaded Exo did not induce apoptosis. Increased apoptosis in MDA-MB-231 cells was detected in the A15-Exo/Dox-treated group in comparison with the Dox-treated group. The Cho-miR159-treated and A15-Exo/Cho-miR159-treated groups also showed approximately 28.26% and 47.15% apoptosis, respectively, indicating that Cho-miR159 promoted apoptosis when delivered by A15-Exo (*p *< 0.05). Furthermore, a synergistic effect of A15-Exo, Cho-miR159, and Dox on apoptosis was observed in MDA-MB-231 cells.

### Quantitative real-time PCR

To evaluate the regulatory effect of miR159 on TCF7 expression, the level of TCF7 mRNA was analyzed by quantitative reverse transcription-PCR (qRT-PCR). As shown in Fig. 4a, the TCF7-expression levels were dramatically higher in Dox- and A15-Exo/Dox-treated MDA-MB-231 cells. Li et al. [51] reported that the Wnt/β-catenin pathway is activated in chemoresistant BC cells. Our results show that the TCF7 expression level in MDA-MB-231 cells increased in response to chemotherapy. After treatment with Cho-miR159, A15-Exo/Cho-miR159, or Co-A15-Exo, the TCF7-expression level substantially declined compared with the Dox and A15-Exo/Dox groups (*p* < 0.05). The qRT-PCR data indicated that co-delivery of Cho-miR159 and Dox by A15-Exo micelles effectively inhibited TCF7 expression.

### Western blotting

To evaluate the regulatory effects of miR159 at the protein level, the protein levels of TCF7 and MYC were analyzed by western blotting. A remarkable decrease in the TCF7 and MYC protein levels was observed in MDA-MB-231 cells in western blot assays performed after introducing miR159 into the cells (Fig. 4d). These results suggest that translation of TCF7 and MYC mRNAs was downregulated in MDA-MB-231 cells by miR-159 introduced by A15-Exo.

### In vitro migration and invasion assays

To study whether Co-A15-Exo suspension can directly stimulate MDA-MB-231 cell migration in vitro, both scratch-wound healing migration and transwell assays were performed. Scratch assays were conducted to evaluate the migration ability. The results shown in Additional file 1: Figure S3 indicate that the Control-, Cho-miR159-, and Dox-treated cells showed complete wound healing after a 24-h incubation, whereas the wounds in cell monolayers treated with A15-Exo, A15-Exo/Cho-miR159, A15-Exo/Dox, and Co-A15-Exo were not completely healed after 24 h, especially in the Co-A15-Exo group. The scratch assay results merely reflected the migratory abilities of the cells. In the A15-Exo group, the wounds were not completely healed, which indicated that A15-Exo could suppress MDA-MB-231 cell motility, but had little effect on MDA-MB-231 cell proliferation (Fig. 4a). Results from transwell matrigel invasion assay showed that Co-A15-Exo significantly inhibited the invasion capacity of MDA-MB-231 cells (Additional file 1: Figure S4). The number of invasive cells from Exo and A15-Exo treatment cells were 110 ± 12.6 and 65 ± 7.90, respectively (*p* < 0.05).

### Biodistribution

**Fig. 5:**
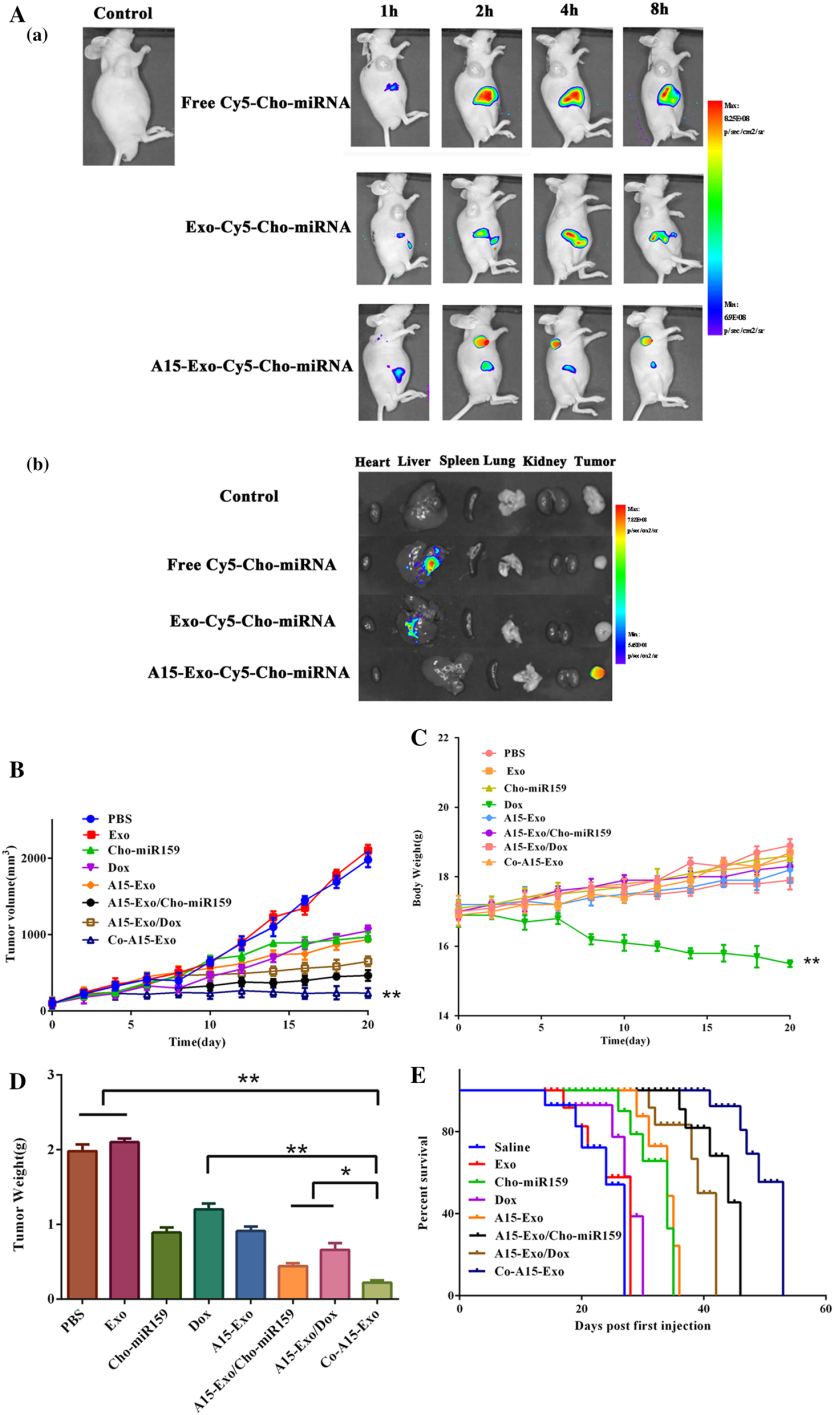
Biodistribution and antitumor efficacy of Co-A15-Exo in vivo. **A** In vivo imaging of Cy5-Cho-miRNA loaded Exo in MDA-MB-231 tumor-bearing nude mice after tail vein injection of free Cy5-Cho-miRNA, Exo-Cy5-Cho-miRNA, or A15-Exo-Cy5-Cho-miRNA. (a) Images were taken 1 h, 2 h, 4 h, or 8 h after the administration of free Cy5-Cho-miRNA, Exo-Cy5-Cho-miRNA, or A15-Exo-Cy5-Cho-miRNA. (b) Ex vivo imaging of tumor and organs collected at the end of the experiment (8 h post-injection). **B** Tumor growth curves of mice receiving different therapeutic regimens (n = 5, mean ± SD). **C** Body weight changes during treatment. Data are expressed as the mean ± SD (n = 5). **p < 0.01, vs. PBS. **D** The weights of the excised tumor tissues from all groups. Data are expressed as the mean ± SD (n = 5). *p < 0.05 and **p < 0.01 when compared with the indicated groups. **E** Survival rate of MDA-MB-231 tumor-bearing BALB/c nude mice

We evaluated the tumor-targeting effect of A15-Exo in vivo using a tumor xenograft mouse model (i.e., BALB/C-nu mice). Figure 5Aa shows images obtained after live mice were scanned at 1, 2, 4, and 8 h post-drug administration. No specific fluorescence was detected in tumor tissues from untreated mice. At 2 h after administration, a substantial accumulation of Cy5 fluorescence was observed by whole-mouse imaging in the tumor areas in the A15-Exo-Cy5-Cho-miRNA group, whereas Cy5 fluorescence was observed in the liver or kidney in the group injected with Exo-Cy5-Cho-miRNA or free Cy5-Cho-miRNA. Overall, these results indicate that A15-Exo served as a highly efficient drug-delivery vehicle for targeted intracellular miRNA delivery. At 8 h post-injection, the organs and tumors were harvested, and fluorescence images were captured (Fig. 5Ab). The images showed that most Cho-Cy5-miRNA delivered by A15-Exo accumulated in the tumor, whereas free Cy5-Cho-miRNA or Exo-Cy5-Cho-miRNA only accumulated in the liver. Collectively, these data suggest that A15-Exo performed well in targeted miRNA delivery to malignant tumors.

### Tumor suppression by Co-A15-Exo

We further evaluated the in vivo therapeutic efficacy of Co-A15-Exo against MDA-MB-231 cells in a xenografted-nude mouse model. The tumor volumes and body weights of the mice were monitored at regular intervals (Fig. 5B, C). Overall, the tumor volumes increased more in the Dox groups than in the Co-A15-Exo group (*p* < 0.05). At day 20 after drug administration, the sizes of the tumors in the Co-A15-Exo group were 4.13-, 4.47-, 3.98-, 1.97-, and 2.77-fold smaller than that in the Cho-miR159, Dox, A15-Exo, A15-Exo/Cho-miR159, A15-Exo/Dox groups, respectively, which confirms the synergistic effect of A15-Exo, Cho-miR159, and Dox against MDA-MB-231 solid tumors in vivo. Notably, the overall inhibitory rates in terms of the tumor volumes were 49.5, 55.6, 53.7, 80.6, 70.7, and 92.8% in the Dox, A15-Exo, Cho-miR159, A15-Exo/Cho-miR159, A15-Exo/Dox and Co-A15-Exo groups, respectively, which strongly demonstrates the potent synergism achieved with A15-Exo mediated co-delivery. These in vivo results corroborated the in vitro cellular assay data. Except for that found in the free Dox group, the mean body weight was not significantly different among the other treatment groups (Fig. 5D), indicating that the systemic toxicity of our therapeutic regimen was low.

In addition to changes in the tumor size, the survival of treated mice was recorded. As shown in Fig. 5E, the mean survival duration of mice in the saline group was 27 days vs. 53 days in the Co-A15-Exo group, 30 days in the Dox group, and 28 days in the Exo group, indicating that Co-A15-Exo effectively inhibited tumor growth and increased the survival of tumor-bearing mice.

### Histology and immunohistochemistry analysis

**Fig. 6:**
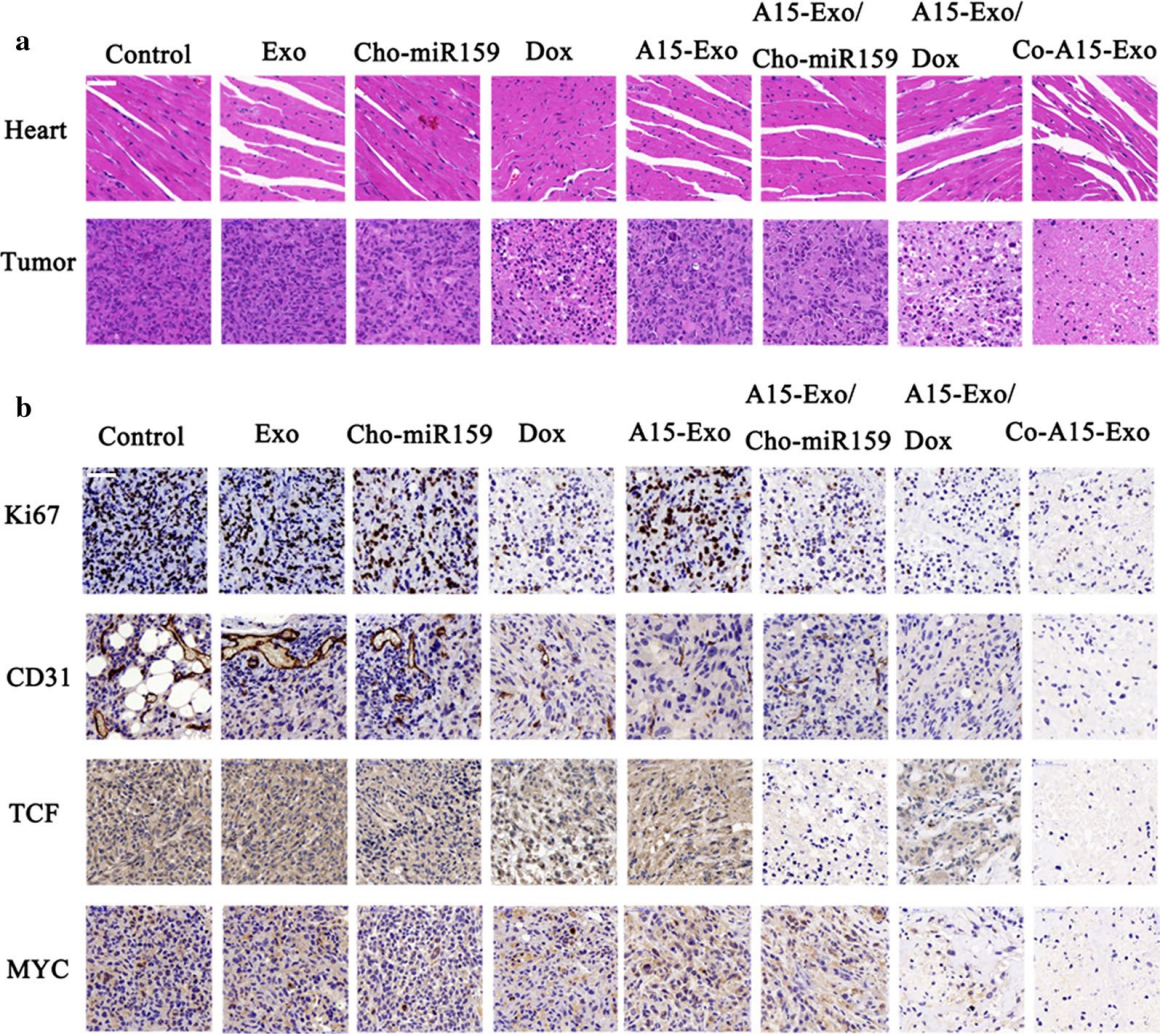
Immunohistochemistry. **a** Representative H&E staining of tumor and heart tissues. Magnification, × 400. **b** IHC analysis of Ki67, CD31, TCF, and MYC expression in tumor sections. Scale bar, 500 μm

Hematoxylin and eosin (H&E) staining showed that damage to cardiac muscle occurred only in the free Dox-treated group, and no obvious cardiac tissue damage was observed in any of the Exo-treated groups or Cho-miR159-treated groups, compared with the control group. As shown in Fig. 6a, our results demonstrate that Dox delivered by Exo remarkably reduced cardiotoxicity compared with free Dox. H&E staining of tumor tissue indicated that necrosis was more evident in Co-A15-Exo treated tumors than in groups treated with PBS, Exo, Cho-miR159, Dox, A15-Exo, A15-Exo/Cho-miR159, or A15-Exo/Dox. A small amount of necrosis was observed in tumor tissues from the PBS- and Exo-treated groups (Fig. 6a). Some necrotic areas were observed in tumor tissues from the A15-Exo-treated group. This finding may be explained by the observation that A15 binds to α_v_β_3_ in an RGD-dependent manner and plays a role in exosome-mediated tumor suppression as a modulating factor of the tumor microenvironment [16].

We analyzed the microvessel density (MVD) marker CD31 in the tumor sections by performing immunohistochemical (IHC) staining to evaluate neovascularization in tumor xenografts. As shown in Fig. 6b, tumor sections in the control-, Exo-, and Cho-miR159-treated groups showed relatively abundant MVD formation. Following Dox, A15-Exo, A15-Exo/Cho-miR159, A15-Exo/Dox, or Co-A15-Exo treatment, the MVD level substantially decreased in the tumor section, as a result of the effective anti-angiogenic activity of A15-Exo.

Immunohistochemical staining against Ki67 can be used to assess the proliferative index of a tumor [52]. As shown in Fig. 6b, we found that Ki67-positive cells were significantly more abundant in the control-, Exo-, Cho-miR159-, and A15-Exo-treated tumors than in the Dox-, A15-Exo/Cho-miR159-, A15-Exo/Dox-, and Co-A15-Exo-treated tumors. These observations suggest that Dox, A15-Exo/Cho-miR159, A15-Exo/Dox, and Co-A15-Exo effectively abrogated the tumor-proliferation rate, especially in the Co-A15-Exo group. The Ki67 level was not significantly altered in the A15-Exo group, which indicated that A15-Exo had no effect on tumor cell proliferation. This finding was in accordance with the in vitro results of the cell-viability and apoptosis assays. The results may be attributable to A15-Exo mediated tumor suppression, whereby A15 modulates the tumor microenvironment in vivo.

As shown in Fig. 6b, Cho-miR159 loading by A15-Exo significantly reduced TCF7 and MYC expression in tumors, with decreased tumor cell proliferation and increased apoptosis, which were still observed in the Co-A15-Exo group. We also observed that TCF7 and MYC expression increased in the Dox and A15-Dox groups, in accordance with our in vitro results. The above data indicate that the Co-A15-Exo-induced inhibitory effect on tumor growth was associated with the co-delivery of Dox and Cho-miR159 via A15-Exo, which could target and interfere with the ligand of integrin α_v_β_3_.

## Discussion

Herein, we report a series of experiments performed to determine the production, targeting effects, and therapeutic effects of A15-Exo loaded with a chemotherapeutic drug and cholesterol-modified miRNA. We isolated A15-Exo with high efficiency by PMA-mediated protein kinase C (PKC) activation of THP-1 cells. The A15 targeting mode provided the Exo with more powerful and precise targeting properties, which were confirmed in vitro and in vivo. By delivering chemotherapeutic drugs and miRNAs, these functional Exo enabled effective tumor therapy in vivo (Fig. 5B). Data from a previous study showed that the A15 gene copy number was increased in cell lines derived from mammary tumors; however, that increase did not correlate with the mRNA level. The study also showed that A15 is associated with cancer, genomic rearrangements, and altered behavior found in tumor cells [53]. Our experimental data showed that Exo derived from continuous PKC activation in monocyte-derived macrophages suppressed tumor growth in vivo. However, A15-Exo did not inhibit MDA-MB-231 cell proliferation and did not promote apoptosis in MDA-MB-231 cells (Fig. 4a, c), although A15-Exo could inhibit MDA-MB-231 cell migration (Additional file 1: Figures S3, S4).

It was previously reported that the RGD integrin-binding sequence of A15 (as the ligand for integrin α_v_β_3_) could decrease ovarian cancer cell adhesion and motility by interfering with the function of α_v_β_3_ [54]. However, other mechanisms that enhance cell–cell adhesion involved in A15-mediated motility have also been reported [55]. There were reports that A15 has the potential for involvement in cell migration at several levels because of its proteolytic activity [56, 57]. Taken together, the results of migration and invasion assays in vitro (Additional file 1: Figures S3, S4) support the notion that A15-Exo or A15-Exo drug loading group (A15-Exo/Cho-miR159, A15-Exo/Dox, and Co-A15-Exo) significantly induce tumor cell migration and invasiveness.

To improve the antitumor activity of Exo nanovesicles, we utilized combinations of nanovesicles containing targeting molecules for co-delivery of miR159 and the chemotherapeutic drug Dox into TNBC cells, in vitro and in vivo. A15 is the ligand for integrin α_v_β_3_. It was reported that A15 could interfere with the function of α_v_β_3_ [20]. Specifically, α_v_β_3_ integrins (identified as markers of angiogenic vascular tissues) are intimately involved in the proliferation and movement of vascular endothelial cells. Blocking α_v_β_3_ integrins could suppress tumor angiogenesis, supporting the potential importance of the function of these integrins. The antitumor effect in vivo suggest that (1) the antitumor effect of Co-A15-Exo was significantly greater than those for A15-Exo/Cho-miR159 and A15-Exo/Dox or Dox, and (2) the presence of A15 on the surface of the Exo is crucial for the targeted delivery of Dox and Cho-miR159 to malignant tumors.

Our findings also showed that the yield of A15-Exo derived from monocyte-derived macrophages was 2.97-fold higher than that of Exo from the same number of monocyte cells. MiRNAs can be secreted through extracellular vesicles and transferred to neighboring or distant cells to modulate cell functions [58, 59]. Wang et al. reported that most miR159 in human serum was detected in an EV-enriched serum fraction after oral administration of miR159 and that miR159 in human serum could inhibit BC cell proliferation [37].

## Conclusion

Here, the data generated in this study provide proof of concept that tumors can be efficiently treated using A15-Exo to co-deliver miRNA and chemotherapeutics via biomimicry. The A15-Exo used in this study expressed appropriate targeting molecules on their surfaces, which may facilitate the development of natural theranostic nanoplatforms with the potential for combined therapy against cancer.

## Methods

### Cell culture

THP-1 cells (human monocytes, ATCC TIB-202) and B16 cells were maintained in RPMI 1640 medium. MDA-MB-231 cells were maintained in Leibovitz L-15 medium. All media were supplemented with 10% fetal bovine serum (FBS) and antibiotics (100 U/mL penicillin and streptomycin). MDA-MB-231 cells were cultured at 37 °C in a humidified CO_2_-free atmosphere, and other mycoplasma-free cells were cultured at 37 °C in a humidified atmosphere containing 5% CO_2_.

### Animals

Animal experiments were conducted in accordance with the National Institutes of Health guide for the care and use of laboratory animals (NIH Publication No. 8023, revised 1978) and were approved by the Research Center for Laboratory Animal of The Second Military Medical University of China.

### Isolation of Exo

THP-1 cells were incubated with or without PMA in serum-free medium in 75-cm^2^ culture flasks for 24 h. The conditioned medium was sequentially centrifuged at 300×*g* for 10 min, 1200×*g* for 20 min, and 10,000×*g* for 30 min to remove cellular debris, after which it was filtered through a 0.22-μm-pore filter (Merck Millipore, Billerica, Massachusetts, US) to separate shed vesicles from the microvesicles [60]. Subsequently, the Exo were pelleted by ultracentrifugation at 100,000×*g* for 70 min at 4 °C, using a Type P70AT rotor (CP80WX; Hitachi Koki Co., Ltd., Tokyo, Japan) and resuspended in PBS. Pellets were suspended in 1 mL PBS and centrifuged for 70 min at 100,000×*g*, 4 °C in a tabletop ultracentrifuge, using a P50A3-0068 rotor in a CP80WX preparative centrifuge (Hitachi Koki Co., Ltd.). Pellets were suspended in PBS and stored at − 80 °C for further experiments. Protein concentrations were measured with a Micro BCA Protein Assay Kit (Pierce, Rockford, IL, USA).

### Differentiation of human monocyte-derived macrophages

Flow cytometric measurements were employed to investigate the expression of the cell surface markers CD11b and A15. THP-1 cells were seeded in a 24-well plate at a density of 3 × 10^5^ cells per well in the presence of 25, 50, or 100 nM PMA. After differentiation for 24 h, the cells were harvested and washed twice with fluorescence-activated cell sorting (FACS) buffer. THP-1 monocytes and differentiated macrophages were double-stained with a phycoerythrin-conjugated anti-CD11b-PE antibody (BioLegend, San Diego, CA, USA) and an APC-conjugated anti-A15 antibody (Miltenyi Biotec, Bergisch Gladbach, Germany). To fully differentiate the THP-1 cells and exclude any direct effects of PMA (50 ng/mL), we washed the PMA-differentiated THP-1 cells with PBS and cultured them for 1–5 additional days, after which the cells were stained with the APC-conjugated anti-A15 antibody. Flow cytometry was performed to analyze the surface expression of A15 of each sample, using a FACSCalibur instrument (BD Bioscience, San Jose, CA, USA).

### Characterization of Exo and A15-Exo

Exo, A15-Exo were purified and diluted in double-distilled water to eliminate the effect of PBS crystallization, fixed with 1% glutaraldehyde, applied onto a carbon-coated copper grid, stained with 1% phosphotungstic acid, and allowed to air dry. A transmission electron microscope (Tecnai G2 spirit; BioTWIN, Hillsboro, OR, USA) was employed to observe the specimens at 120 kV.

The concentrations and sizes of Exo and A15-Exo were determined by recording and analyzing the Brownian motion of particles using a NanoSight NS300 system and Nanoparticle Tracking Analysis software (Malvern Instruments, Malvern, United Kingdom), according to the manufacturer’s protocol. The mean size and size-distribution data were captured and analyzed using the NTA 3.2 Analytical Software Suite. All procedures were performed at room temperature.

### Preparation of Dox-loaded A15-Exo (A15-Exo/Dox), Cho-miR159-loaded A15-Exo (A15-Exo/Cho-miR159), Dox, and Cho-miR159-Loaded A15-Exo (Co-A15-Exo)

Dox packaging into A15-Exo (A15-Exo/Dox) was performed by mixing variable concentrations of Dox (100, 200, 400, 800, or 1000 μg/mL) with Exo in triethylamine solution overnight.

To evaluate the drug-release behavior of A15-Exo/Dox, we determined the in vitro Dox-release profile from A15-Exo at pH 7.4 (physiological environment) and at pH 5.0 (late endosomal and lysosomal environments).

Exo were loaded with the indicated concentrations of Cho-miR159 in PBS by incubation at 37 °C for 90 min, with shaking at 500 rpm. Cy5-labeled Cho-miRNA was used to measure the loading efficiency of Cho-miR159 in Exo and cellular internalization. The loading efficiency of Cho-miRNA in Exo was estimated by directly measuring Cy5 fluorescence in the Cho-miRNA-Exo pellet. In the same way, the loading efficiency of Dox was measured using a Modulus™ single-tube multimode reader (Turner BioSystems, USA). Dox and Cho-miR159-loaded A15-Exo (Co-A15-Exo) were prepared by adding an appropriate amount of Cho-miR159 to A15-Exo/Dox, followed by incubation at 37 °C for 90 min with shaking at 500 rpm. Unloaded Cho-miR159 or Dox were removed by ultracentrifugation for 70 min at 100,000×*g*, 4 °C in a tabletop ultracentrifuge using a P50A3-0068 rotor in a CP80WX preparative centrifuge (Hitachi Koki Co., Ltd.).

The loading effect was also evaluated by assessing A15-Exo and Cy5-Cho-miR159/Dox colocalization using confocal laser-scanning microscopy. Exosomes were labeled using the green lipophilic fluorescent dye PKH67 [61–63] (Sigma-Aldrich, St Louis, Mo). In addition, based on the manufacturer’s protocol and our previous research [64], we standardized the optimal dye and exosome concentrations for experimental purposes. Briefly, 10 μM of PKH67 dye (diluted with Diluent C (Sigma-Aldrich)) was added to the exosome samples in 100 μL PBS, and incubated for 5 min at 37 °C, and labeling was stopped by the addition of Exo-depleted FBS. Free dye was removed by ultracentrifugation for 70 min at 100,000×g, 4 °C in a tabletop ultracentrifuge using a P50A3-0068 rotor in a CP80WX preparative centrifuge (Hitachi Koki Co., Ltd.). They were then visualized by confocal laser scanning microscopy (Nikon, Shinagawa, Tokyo, Japan).

### Cellular uptake of Exo

α_v_β_3_-positive and -negative BC cells (MDA-MB-231 and MCF-7) were used as models for studying Exo uptake. MDA-MB-231 and MCF-7 cells were incubated with PKH67-labeled Exo (100 μg/mL) for 4 h. The confocal images were recorded continuously with a confocal laser-scanning microscope (Nikon) after adding the A15-Exo or Exo suspension.

To investigate whether A15-Exo could bind to α_v_β_3_ integrin-positive cancer cells, A15-Exo or Exo (100 μg/mL) were labeled with PKH67 and cultured with α_v_β_3_-positive MDA-MB-231 and melanoma B16 cells. Exo-uptake experiments were performed under serum-free conditions. Cells were detached, and the remaining surface-associated Exo were removed by trypsinization. Cells were washed in PBS, resuspended in PBS, and analyzed on a FACSCalibur instrument integrated with Cell-Quest software (BD Biosciences).

### Cellular uptake of Cho-miRNA and Dox

To investigate the cellular uptake of Cho-miRNA and Dox mediated by A15-Exo or Exo, MDA-MB-231 and B16 cells were incubated for 4 h with Cy5-miRNA, Cy5-Cho-miRNA, Exo-Cy5-Cho-miRNA, A15-Exo-Cy5-Cho-miRNA, Dox, or A15-Exo/Dox. Next, the culture medium was removed, and the cells were washed twice with PBS and trypsinized. The cells were then suspended in PBS, and the cellular uptake of Cy5-miRNA and Dox by Exo and A15-Exo was determined using a FACSCalibur instrument (BD Biosciences, UK). The experiment was repeated 3 times.

### Confocal microscopy

Confocal fluorescence microscopy was performed to assess the intracellular trafficking of Exo, A15-Exo, miR159, and Dox. MDA-MB-231 and B16 cells that were grown on glass coverslips in a 24-well plate were incubated with Exo, A15-Exo, Dox, A15-Exo/Dox, Cy5-Cho-miRNA, A15-Exo/Cy5-Cho-miRNA, or A15-Exo/Dox/Cy5-Cho-miRNA for 4 h. Following incubation, the cells were washed three times with PBS and fixed in paraformaldehyde for 30 min. Localization of Exo, A15-Exo, miRNA, and Dox in cells was visualized using a confocal microscope (Nikon), with identical settings used for each confocal study.

### Cell-viability assay

A Cell Counting Kit-8 (CCK-8, Dojindo, Japan) cell-viability assay was performed on MDA-MB-231 cells to assess the cytotoxic effects of Co-A15-Exo. Firstly, MDA-MB-231 cells were seeded overnight in 96-well plates at a density of 8 × 10^3^ cells/well. Cells were washed three times with serum-free media and incubated for 4 h with Exo, Cho-miR159, Dox, A15-Exo, A15-Exo/Cho-miR159, A15-Exo/Dox, or Co-A15-Exo (2 mM Dox and 500 fmoL/mL Cho-miR159). Subsequently, the medium was replaced with fresh cell culture medium, and the cells were incubated at 37 °C for an additional 48 h. CCK-8 solution was added according to the manufacturer’s protocol, and cell viability was calculated as the ratio of the absorbance of test and control wells. A microplate reader (ThermoFisher Scientific, Waltham, MA) was used to determine the optical density at 450 nm. Each treatment condition was repeated in quintuplicate, and all data are expressed as the mean ± SD.

### Apoptosis assay

To determine the rate of apoptosis, MDA-MB-231 cells were first seeded overnight in 12-well plates at a density of 1.5 × 10^5^ cells/well. After treatment with Exo, Cho-miR159, Dox, A15-Exo, A15-Exo/Cho-miR159, A15-Exo/Dox, or Co-A15-Exo (2 mM Dox and 500 fmoL/mL Cho-miR159), the cells were trypsinized, harvested, washed with PBS, and stained with Annexin V-APC and PI according to the manufacturer’s protocol (eBioscience, San Diego, CA). At least 10,000 cells from each sample were analyzed using a FACSCalibur flow cytometer (BD Biosciences, UK). Untreated cells were used as controls to determine the background rate of apoptosis.

### In vitro migration and invasion assays

The scratch-wound cell migration assay was performed, as described previously [65]. Briefly, cells were seeded at a density of 3 × 10^5^ cells/mL (applied in 70 μL/well (Ibidi Culture-Inserts; Ibidi, Martinsried, Germany). The Ibidi Culture-Insert enabled the formation of a well-defined gap, without wounding the cell monolayer. After being cultured for 24 h, the cell monolayer was optically confluent. The cells were then washed twice with medium and then provided serum-free medium. Then, Exo, Cho-miR159, Dox, A15-Exo, A15-Exo/Cho-miR159, A15-Exo/Dox, or Co-A15-Exo (2 mM Dox and 500 fmoL/mL Cho-miR159) were added to a corner of each well, and the cells were further cultured. The scratch was monitored by taking images at 0 and 24 h using a microscopy (Olympus IX73, Tokyo, Japan).

For the transwell assay, MD-MB-231 cells with or without Exo, Cho-miR159, Dox, A15-Exo, A15-Exo/Cho-miR159, A15-Exo/Dox, or Co-A15-Exo (2 mM Dox and 500 fmoL/mL Cho-miR159) treatment were resuspended in L-15/0.1% BSA and seeded into the upper chamber of transwell 24-well plates (Corning, Corning, NY, USA) with 8 μm pore filters at a density of 1 × 10^4^ cells/well (three replicates per group) were suspended in L-15/0.1% BSA and seeded into the upper chamber of transwell 24-well plates (Corning, Corning, NY, USA) with 8 μm pore filters. Then the lower chamber was added with L-15/20% FBS. After incubation for 24 h at 37 °C, the cells remained on the upper surface of the filter membranes were removed. The migrated cells of the lower surface were fixed with 4% paraformaldehyde, and staining with 0.1% crystal violet solution for 15 min. The level of migration was captured with a microscopy (Olympus IX73, Tokyo, Japan).

### qRT-PCR experiments

The TCF7 mRNA level was analyzed by qRT-PCR. Total RNA was extracted using the TRIzol^®^ reagent (Invitrogen, Carlsbad, CA, USA), according to the manufacturer’s protocol. qRT-PCR was performed using the SuperScript^®^ III Platinum^®^ One-Step qRT-PCR Kit, according to the protocol provided (Invitrogen). All data were analyzed, using GAPDH mRNA expression as an internal standard. Primers with the following sequences were used: TCF7, 5′-TCCGCCTTCAATCTGCTCAT-3′ (forward) and 5′-AACTTGCTTCTGGCTGATGTCC-3′ (reverse); GAPDH, 5′-GGAAGCTTGTCATCAATGGAAATC-3′ (forward) and 5′-TGATGACCCTTTTGGCTCCC-3′ (reverse).

### Western blotting

Cell lysates were prepared by freezing and thawing four times, followed by centrifugation at 15,000×*g* for 15 min to remove cell debris. Exo and cell lysates (5 mg of protein) were reduced with 0.1 M dithiothreitol and heated at 95 °C for 3 min. The samples were then subjected to 10% sodium dodecyl sulfate-polyacrylamide gel electrophoresis and electrophoretically transferred to a polyvinylidene fluoride membrane. The membrane was blocked with Blocking One solution (Nacalai Tesque, Kyoto, Japan) for 30 min. The membrane was subsequently probed with primary antibodies for 1 h at room temperature. The membranes were washed and incubated with secondary horseradish peroxidase-conjugated antibodies for 30 min at room temperature. The following primary antibodies were used: a rabbit anti-streptavidin antibody (Sigma-Aldrich, Germany), a mouse anti-Alix antibody (BD Biosciences, San Jose, CA, USA), an anti-TCF7 antibody (Cell Signaling Technology, Danvers, MA, USA), and an anti-MYC antibody (Cell Signaling Technology, Danvers, MA, USA). Bands were visualized using an Enhanced Chemiluminescence Kit (Millipore, Bedford, MA, USA). Images were obtained using a GE ImageQuant Las 4000 mini imager (GE, Fairfield, CT, USA).

### In vivo targeting and biodistribution

To investigate the potential of A15-Exo to specifically deliver Dox to tumor tissues in vivo, we established an MDA-MB-231 tumor-bearing nude mouse model. PBS, naked Cy5-Cho-miRNA, Exo/Cy5-Cho-miRNA, or A15-Exo/Cy5-Cho-miRNA were injected via the tail vein at a single miRNA dose of 1 mg/kg (250 μL solution). The mice were then scanned at 1, 2, 4, 8 h post-injection using the Bio-Real Quick View 3000 imaging system (Bio-Real Sciences, Austria). Mice were sacrificed at 8 h after administration, after which the tumors, heart, liver, spleen, lungs, and kidneys were isolated, washed in saline, and immediately imaged. The exposure time was set to 1 s/image. Images were analyzed using Living Imaging software (Bio-Real Sciences).

### In vivo anti-tumor effects

A xenograft-tumor model was generated by injecting 0.1 mL of a MDA-MB-231 cell suspension (1 × 10^7^ cells) into the right flank of male BALB/c nude mice. Mice bearing established MDA-MB-231 tumors (~ 0.1 cm^3^) were randomly sorted into eight groups, and the groups were treated as follows: (i) PBS, as a control; (ii) Exo; (iii) Cho-miR159; (iv) Dox; (v) A15-Exo; (vi) A15-Exo/Cho-miR159; (vii) A15-Exo/Dox; (viii) Co-A15-Exo (a Dox equivalent of 5 mg/kg and a Cho-miR159 equivalent of 0.1 nmoL/kg). The drugs were injected weekly through the tail vein for 5 consecutive weeks. The mice were weighed, and the tumors were measured with a caliper every 4 days. Tumor volumes were calculated using the following equation: tumor volume = length × width^2^/2. The mouse weights and survival rates were also recorded. The tumor-inhibition rate (TIR) was calculated using the following equation:

$${\text{TIR}} = \left( { 1{-}\left[ {{\text{W}}_{\text{test}} {-}{\text{W}}_{\text{initial}} } \right]/\left[ {{\text{W}}_{\text{saline}} {-}{\text{W}}_{\text{initial}} } \right]} \right) \times 100\% ,$$

where W_test_ is the final mean tumor weight for each tested group, W_initial_ is the initial mean tumor weight for each tested group, and W_saline_ refers to the final mean tumor weight of the saline group.

### Histology and immunohistochemistry

After the mice were sacrificed, the hearts and tumors were collected and fixed in 4% paraformaldehyde for 24 h and subsequently embedded in paraffin. Tissue sections (5 μm) were stained with H&E. IHC staining on tumor tissues harvested from treated mice was performed using an automated immunostainer (Leica, Bond-III, Leica, Buffalo Grove, IL) with the primary polyclonal antibodies against Ki67 and mouse CD31 (Abcam, Cambridge, MA). The stained tissue sections were analyzed using the Aperio ScanScope Image Analysis System (Aperio, Vista, CA). TCF7 (C63D9) and MYC (D84C12) antibodies were obtained from Cell Signaling Technology. Ki67 antibodies were obtained from Dako (MIB-1; Carpinteria, CA, USA).

### Statistical analysis

The mean value ± SD was determined for each treatment group, and each value presented represents the mean of at least three replicate experiments for each group. Non-parametric testing was performed using IBM SPSS statistical-analysis software to assess the significance of differences between, two groups. *p < 0.05 and **p < 0.01.

## Supplementary information

**Additional file 1: Figure S1.** THP-1 monocyte differentiation induced by PMA. (A) Quantitative analysis expression of CD11b and A15 in THP-1 monocytes and differentiated macrophages for different treated concentration of PMA. (B) a)Flow cytometry analysis of A15 expression in THP-1 cells treated with PMA (50 ng/mL) for different times; b) mean fluorescence intensity of A15 expression in THP-1 cells treated with PMA (50 ng/mL) for different times. **Figure S2.** Confocal laser-scanning microscopy (CLSM) images of MDA-MB-231 and MCF-7 incubated with PKH7 labled Exo or A15-Exo at 37 °C for 4 h. **Figure S3.** Wound healing assay of MDA-MB-231 cells incubated with Co-A15-Exo displayed arrested healing/closing of the scratch. (representative pictures from 3 repeated experiments) Scale bar: 100 μm. **Figure S4.** The migratory ability of MDA-MB-231 receiving different treatments was further confirmed by the transwell assay and quantitative analysis of the migrated cells. n = 3 per group. Scale bar, 100 μm.

## Data Availability

All data generated or analysed during this study are included in this published article and its additional information files.

## Abbreviations

A15: a disintegrin and metalloproteinase 15
A15-Exo: Exo loaded with A15
A15-Exo/Dox: A15 Exo loaded with Dox
APC: allophycocyanin
BC: breast cancer
Cho-miR159: cholesterol-modified miRNA 159
Co-A15-Exo: A15 Exo loaded with Cho-miR159 and Dox
Dox: doxorubicin
EN-2: engrailed-2
Exo: exosomes
H&E: hematoxylin and eosin
IHC: immunohistochemical
miRNA: microRNA
mRNA: messenger RNA
MVD: microvessel density
NCOA6: nuclear receptor coactivator 6
PBS: phosphate-buffered saline
PI: propidium iodide
PKC: protein kinase C
PMA: phorbol 12-myristate 13-acetate
qRT-PCR: quantitative reverse transcriptase-polymerase chain reaction
RGD: Arg-Gly-Asp
siRNA: short interfering RNA
TCF7: transcription factor 7
TIR: tumor-inhibitory rate
TNBC: triple-negative breast cancer

## References

[1] Foulkes WD, Smith IE, Reis-Filho JS (2010). Triple-negative breast cancer. N Engl J Med.

[2] Stevens KN, Vachon CM, Couch FJ (2013). Genetic susceptibility to triple-negative breast cancer. Cancer Res.

[3] Sparano JA (2015). Defining a role and predicting benefit from platinum-based therapy in breast cancer: an evolving story. J Clin Oncol.

[4] Tung NM, Winer EP (2015). Tumor-infiltrating lymphocytes and response to platinum in triple-negative breast cancer. J Clin Oncol.

[5] Cancello G, Bagnardi V, Sangalli C, Montagna E, Dellapasqua S, Sporchia A (2015). Phase II study with epirubicin, cisplatin, and infusional fluorouracil followed by weekly paclitaxel with metronomic cyclophosphamide as a preoperative treatment of triple-negative breast cancer. Clin Breast Cancer.

[6] Torrisi R, Balduzzi A, Ghisini R, Rocca A, Bottiglieri L, Giovanardi F (2008). Tailored preoperative treatment of locally advanced triple negative (hormone receptor negative and HER2 negative) breast cancer with epirubicin, cisplatin, and infusional fluorouracil followed by weekly paclitaxel. Cancer Chemother Pharmacol.

[7] van der Pol E, Coumans FA, Grootemaat AE, Gardiner C, Sargent IL, Harrison P (2014). Particle size distribution of exosomes and microvesicles determined by transmission electron microscopy, flow cytometry, nanoparticle tracking analysis, and resistive pulse sensing. J Thromb Haemost.

[8] Mathieu M, Martin-Jaular L, Lavieu G, Théry C (2019). Specificities of secretion and uptake of exosomes and other extracellular vesicles for cell-to-cell communication. Nat Cell Biol.

[9] Li I, Nabet BY (2019). Exosomes in the tumor microenvironment as mediators of cancer therapy resistance. Mol Cancer.

[10] Yang H, Zhang H, Ge S, Ning T, Bai M, Li J (2018). Exosome-derived miR-130a activates angiogenesis in gastric cancer by targeting C-MYB in vascular endothelial cells. Mol Ther.

[11] Shi M, Sheng L, Stewart T, Zabetian CP, Zhang J (2019). New windows into the brain: central nervous system-derived extracellular vesicles in blood. Prog Neurobiol.

[12] Bunggulawa EJ, Wang W, Yin T, Wang N, Durkan C, Wang Y (2018). Recent advancements in the use of exosomes as drug delivery systems. J Nanobiotechnol.

[13] Sancho-Albero M, Navascués N, Mendoza G, Sebastián V, Arruebo M, Martín-Duque P (2019). Exosome origin determines cell targeting and the transfer of therapeutic nanoparticles towards target cells. J Nanobiotechnol.

[14] Zhuang X, Xiang X, Grizzle W, Sun D, Zhang S, Axtell RC (2011). Treatment of brain inflammatory diseases by delivering exosome encapsulated anti-inflammatory drugs from the nasal region to the brain. Mol Ther.

[15] Jang SC, Kim OY, Yoon CM, Choi DS, Roh TY, Park J (2013). Bioinspired exosome-mimetic nanovesicles for targeted delivery of chemotherapeutics to malignant tumors. ACS Nano.

[16] Zhang D, Lee H, Wang X, Rai A, Groot M, Jin Y (2018). Exosome-mediated small RNA delivery: a novel therapeutic approach for inflammatory lung responses. Mol Ther.

[17] Chen Q, Meng LH, Zhu CH, Lin LP, Lu H, Ding J (2008). ADAM15 suppresses cell motility by driving integrin α5β1 cell surface expression via erk inactivation. Int J Biochem Cell Biol.

[18] Toquet C, Colson A, Jarry A, Bezieau S, Volteau C, Boisseau P (2012). ADAM15 to α5β1 integrin switch in colon carcinoma cells: a late event in cancer progression associated with tumor dedifferentiation and poor prognosis. Int J Cancer.

[19] Ungerer C, Doberstein K, Bürger C, Hardt K, Boehncke WH, Böhm B (2010). ADAM15 expression is downregulated in melanoma metastasis compared to primary melanoma. Biochem Biophys Res Commun.

[20] Lee HD, Koo BH, Kim YH, Jeon OH, Kim DS (2012). Exosome release of ADAM15 and the functional implications of human macrophage-derived ADAM15 exosomes. FASEB J.

[21] Lastres P, Bellon T, Cabañas C, Sanchez-Madrid F, Acevedo A, Gougos A (1992). Regulated expression on human macrophages of endoglin, an Arg-Gly-Asp-containing surface antigen. Eur J Immunol.

[22] Albelda SM, Mette SA, Elder DE, Stewart R, Damjanovich L, Herlyn M (1990). Integrin distribution in malignant melanoma: association of the beta 3 subunit with tumor progression. Cancer Res.

[23] Gingras MC, Roussel E, Bruner JM, Branch CD, Moser RP (1995). Comparison of cell adhesion molecule expression between glioblastoma multiforme and autologous normal brain tissue. J Neuroimmunol.

[24] Natali PG, Hamby CV, Felding-Habermann B, Liang B, Nicotra MR, Di Filippo F (1997). Clinical significance of alpha(v)beta3 integrin and intercellular adhesion molecule-1 expression in cutaneous malignant melanoma lesions. Cancer Res.

[25] Bartel DP (2004). MicroRNAs: genomics, biogenesis, mechanism, and function. Cell.

[26] Di Leva G, Piovan C, Gasparini P, Ngankeu A, Taccioli C, Briskin D (2013). Estrogen mediated-activation of miR-191/425 cluster modulates tumorigenicity of breast cancer cells depending on estrogen receptor status. PLoS Genet.

[27] Li B, Xu WW, Han L, Chan KT, Tsao SW, Lee NPY, Cheung ALM (2017). MicroRNA-377 suppresses initiation and progression of esophageal cancer by inhibiting CD133 and VEGF. Oncogene.

[28] Png KJ, Halberg N, Yoshida M, Tavazoie SF (2011). A MicroRNA regulon that mediates endothelial recruitment and metastasis by cancer cells. Nature.

[29] Zheng K, Zhou X, Yu J, Li Q, Wang H, Li M (2016). Epigenetic silencing of miR-490-3p promotes development of an aggressive colorectal cancer phenotype through activation of the Wnt/β-catenin signaling pathway. Cancer Lett.

[30] O’Bryan S, Dong S, Mathis JM, Alahari SK (2017). The roles of oncogenic miRNAs and their therapeutic importance in breast cancer. Eur J Cancer.

[31] Didiot MC, Hall LM, Coles AH, Haraszti RA, Godinho BM, Chase K (2016). Exosome-mediated delivery of hydrophobically modified siRNA for huntingtin mRNA silencing. Mol Ther.

[32] Geary RS, Norris D, Yu R, Bennett CF (2015). Pharmacokinetics, biodistribution and cell uptake of antisense oligonucleotides. Adv Drug Deliv Rev.

[33] Park JK, Kogure T, Nuovo GJ, Jiang J, He L, Kim JH (2011). miR-221 silencing blocks hepatocellular carcinoma and promotes survival. Cancer Res.

[34] Wang LL, Chung JJ, Li EC, Uman S, Atluri P, Burdick JA (2018). Injectable and protease-degradable hydrogel for siRNA sequestration and triggered delivery to the heart. J Control Release.

[35] Alvarez-Erviti L, Seow Y, Yin H, Betts C, Lakhal S, Wood MJ (2011). Delivery of siRNA to the mouse brain by systemic injection of targeted exosomes. Nat Biotechnol.

[36] Bryniarski K, Ptak W, Jayakumar A, Püllmann K, Caplan MJ, Chairoungdua A (2013). Antigen-specific, antibody-coated, exosome-like nanovesicles deliver suppressor T-cell microRNA-150 to effector T cells to inhibit contact sensitivity. J Allergy Clin Immunol.

[37] Chin AR, Fong MY, Somlo G, Wu J, Swiderski P, Wu X (2016). Cross-kingdom inhibition of breast cancer growth by plant miR159. Cell Res.

[38] Arce L, Yokoyama NN, Waterman ML (2006). Diversity of LEF/TCF action in development and disease. Oncogene.

[39] Bilir B, Kucuk O, Moreno CS (2013). Wnt signaling blockage inhibits cell proliferation and migration, and induces apoptosis in triple-negative breast cancer cells. J Transl Med.

[40] Lehmann BD, Bauer JA, Chen X, Sanders ME, Chakravarthy AB, Shyr Y (2011). Identification of human triple-negative breast cancer subtypes and preclinical models for selection of targeted therapies. J Clin Invest.

[41] Johnson JP, Kumar P, Koulnis M, Patel M, Simin K (2014). Crucial and novel cancer drivers in a mouse model of triple-negative breast cancer. Cancer Genom Proteom.

[42] Yang L, Wu X, Wang Y, Zhang K, Wu J, Yuan YC (2011). FZD7 has a critical role in cell proliferation in triple negative breast cancer. Oncogene.

[43] Yao C, Liu J, Wu X, Tai Z, Gao Y, Zhu Q (2016). Reducible self-assembling cationic polypeptide-based micelles mediate co-delivery of doxorubicin and microRNA-34a for androgen-independent prostate cancer therapy. J Control Release.

[44] Gong C, Hu C, Gu F, Xia Q, Yao C, Zhang L (2017). Co-delivery of autophagy inhibitor ATG7 siRNA and docetaxel for breast cancer treatment. J Control Release.

[45] Schwende H, Fitzke E, Ambs P, Dieter P (1996). Differences in the state of differentiation of THP-1 cells induced by phorbol ester and 1,25-dihydroxyvitamin D_3_. J Leukoc Biol.

[46] Yang Z, Xie J, Zhu J, Kang C, Chiang C, Wang X (2016). Functional exosome-mimic for delivery of siRNA to cancer: in vitro and in vivo evaluation. J Control Release.

[47] Qi H, Liu C, Long L, Ren Y, Zhang S, Chang X (2016). Blood exosomes endowed with magnetic and targeting properties for cancer therapy. ACS Nano.

[48] Liu Z, Sun X, Nakayama-Ratchford N, Dai H (2007). Supramolecular chemistry on water-soluble carbon nanotubes for drug loading and delivery. ACS Nano.

[49] Chen P, Wang Z, Zong S, Zhu D, Chen H, Zhang Y (2016). pH-sensitive nanocarrier based on gold/silver core-shell nanoparticles decorated multi-walled carbon nanotubes for tracing drug release in living cells. Biosens Bioelectron.

[50] Ly S, Navaroli DM, Didiot MC, Cardia J, Pandarinathan L, Alterman JF (2017). Visualization of self-delivering hydrophobically modified siRNA cellular internalization. Nucleic Acids Res.

[51] Zhang ZM, Wu JF, Luo QC, Liu QF, Wu QW, Ye GD (2016). Pygo2 activates MDR1 expression and mediates chemoresistance in breast cancer via the Wnt/β-catenin pathway. Oncogene.

[52] Jalava P, Kuopio T, Juntti-Patinen L, Kotkansalo T, Kronqvist P, Collan Y (2006). Ki67 immunohistochemistry: a valuable marker in prognostication but with a risk of misclassification: proliferation subgroups formed based on Ki67 immunoreactivity and standardized mitotic index. Histopathology.

[53] Ortiz RM, Karkkainen I, Huovila AP (2004). Aberrant alternative exon use and increased copy number of human metalloprotease-disintegrin ADAM15 gene in breast cancer cells. Genes Chromosomes Cancer.

[54] Beck V, Herold H, Benge A, Luber B, Hutzler P, Tschesche H (2005). ADAM15 decreases integrin α_v_β_3_/vitronectin-mediated ovarian cancer cell adhesion and motility in an RGD-dependent fashion. Int J Biochem Cell Biol.

[55] Herren B, Garton KJ, Coats S, Bowen-Pope DF, Ross R, Raines EW (2001). ADAM15 overexpression in NIH3T3 cells enhances cell–cell interactions. Exp Cell Res.

[56] Martin J, Eynstone LV, Davies M, Williams JD, Steadman R (2002). The role of ADAM 15 in glomerular mesangial cell migration. J Biol Chem.

[57] Zhong JL, Poghosyan Z, Pennington CJ, Scott X, Handsley MM, Warn A (2008). Distinct functions of natural ADAM-15 cytoplasmic domain variants in human mammary carcinoma. Mol Cancer Res.

[58] Fong MY, Zhou W, Liu L, Alontaga AY, Chandra M, Ashby J (2015). Breast-cancer-secreted miR-122 reprograms glucose metabolism in premetastatic niche to promote metastasis. Nat Cell Biol.

[59] Jiang N, Xiang L, He L, Yang G, Zheng J, Wang C (2017). Exosomes mediate epithelium–mesenchyme crosstalk in organ development. ACS Nano.

[60] Valadi H, Ekström K, Bossios A, Sjöstrand M, Lee JJ, Lötvall JO (2007). Exosome-mediated transfer of mRNAs and microRNAs is a novel mechanism of genetic exchange between cells. Nat Cell Biol.

[61] Morelli AE, Larregina AT, Shufesky WJ, Sullivan ML, Stolz DB, Papworth GD (2004). Endocytosis, intracellular sorting, and processing of exosomes by dendritic cells. Blood.

[62] Sagar G, Sah RP, Javeed N, Dutta SK, Smyrk TC, Lau JS (2016). Pathogenesis of pancreatic cancer exosome-induced lipolysis in adipose tissue. Gut.

[63] Cheng Y, Wang X, Yang J, Duan X, Yao Y, Shi X (2012). A translational study of urine miRNAs in acute myocardial infarction. J Mol Cell Cardiol.

[64] Li Y, Gao Y, Gong C, Wang Z, Xia Q, Gu F (2018). A33 antibody-functionalized exosomes for targeted delivery of doxorubicin against colorectal cancer. Nanomedicine.

[65] Liu WL, Chang JM, Chong IW, Hung YL, Chen YH, Huang WT (2017). Curcumin inhibits LIN-28A through the activation of miRNA-98 in the lung cancer cell line A549. Molecules.

