# Supplementary material for: Functional exosome-mediated co-delivery of doxorubicin and hydrophobically modified microRNA 159 for triple-negative breast cancer therapy
Source: J Nanobiotechnology. 2019 Sep 3;17:93. doi: 10.1186/s12951-019-0526-7 (PMC6721253; doi:10.1186/s12951-019-0526-7)
Supplement: Supplementary file 1 — Additional file 1: Figure S1. THP-1 monocyte differentiation induced by PMA. (A) Quantitative analysis expression of CD11b and A15 in THP-1 monocytes and differentiated macrophages for different treated concentration of PMA. (B) a)Flow cytometry analysis of A15 expression in THP-1 cells treated with PMA (50 ng/mL) for different times; b) mean fluorescence intensity of A15 expression in THP-1 cells treated with PMA (50 ng/mL) for different times. Figure S2. Confocal laser-scanning microscopy (CLSM) images of MDA-MB-231 and MCF-7 incubated with PKH7 labled Exo or A15-Exo at 37 °C for 4 h. Figure S3. Wound healing assay of MDA-MB-231 cells incubated with Co-A15-Exo displayed arrested healing/closing of the scratch. (representative pictures from 3 repeated experiments) Scale bar: 100 μm. Figure S4. The migratory ability of MDA-MB-231 receiving different treatments was further confirmed by the transwell assay and quantitative analysis of the migrated cells. n = 3 per group. Scale bar, 100 μm. [file 12951_2019_526_MOESM1_ESM.docx]

**Additional files**

Functional exosome-mediated co-delivery of doxorubicin and hydrophobically modified microRNA 159 for triple-negative breast cancer therapy

Chunai Gong,^#a, b^ Jing Tian,^# a^ Zhuo Wang,^#a^ Yuan Gao^c^ Xin Wu^a^ Xueying Ding^d^ Lei Qiang^a^ Guorui Li^a^ Zhimin Han^a^ Yongfang Yuan,^*b^ and Shen Gao,^*a^

**1.PMA-induced monocyte differentiation**

**
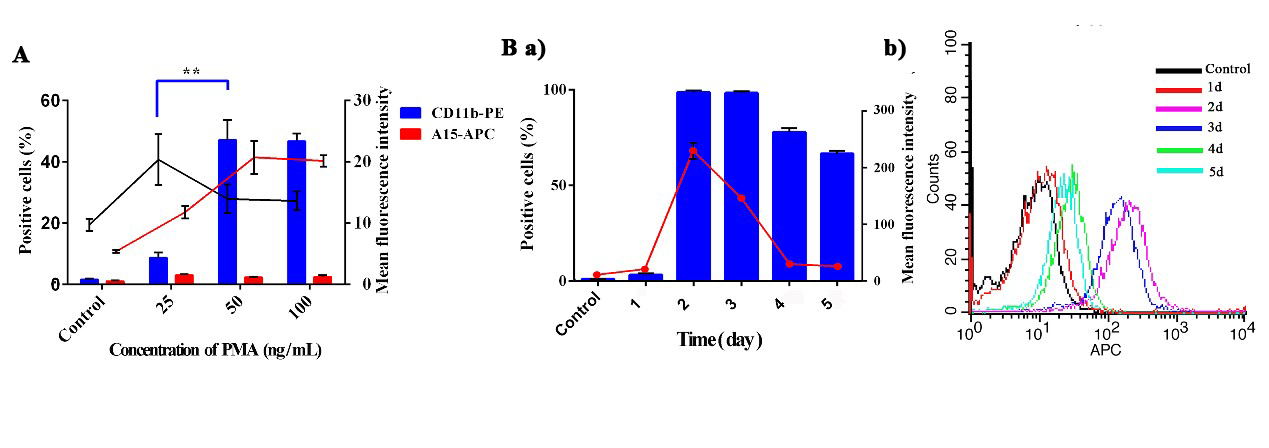
**

Figure S1 THP-1 monocyte differentiation induced by PMA. (A) Quantitative analysis expression of CD11b and A15 in THP-1 monocytes and differentiated macrophages for different treated concentration of PMA. (B) a)Flow cytometry analysis of A15 expression in THP-1 cells treated with PMA (50 ng/mL) for different times; b) mean fluorescence intensity of A15 expression in THP-1 cells treated with PMA (50 ng/mL) for different times;

**2.In vitro targeting of A15-Exo**

**
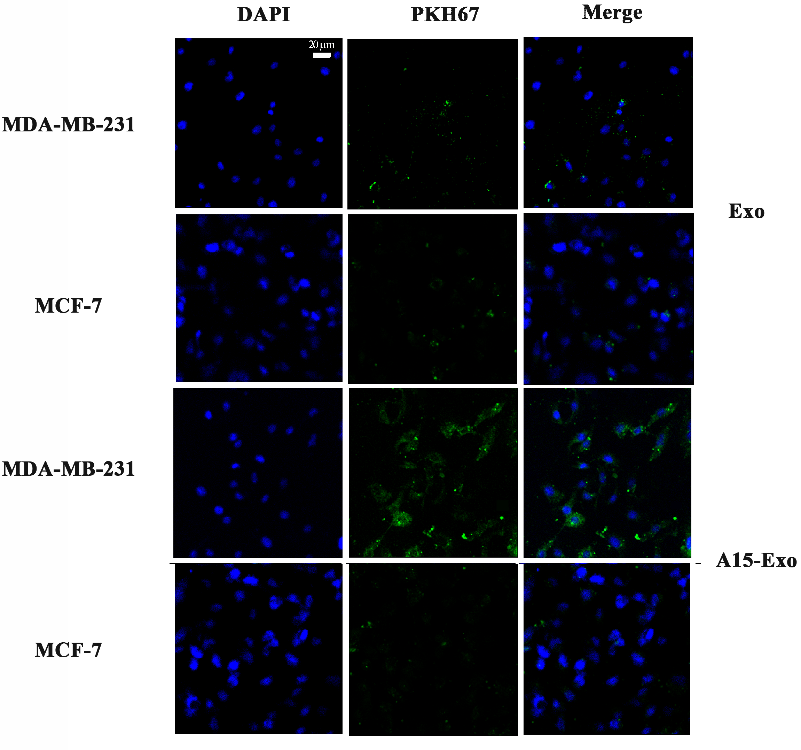
**

Figure S2 Confocal laser-scanning microscopy (CLSM) images of MDA-MB-231 and MCF-7 incubated with PKH7 labled Exo or A15-Exo at 37℃ for 4 h.

**3. Cell-Migration Study**

**
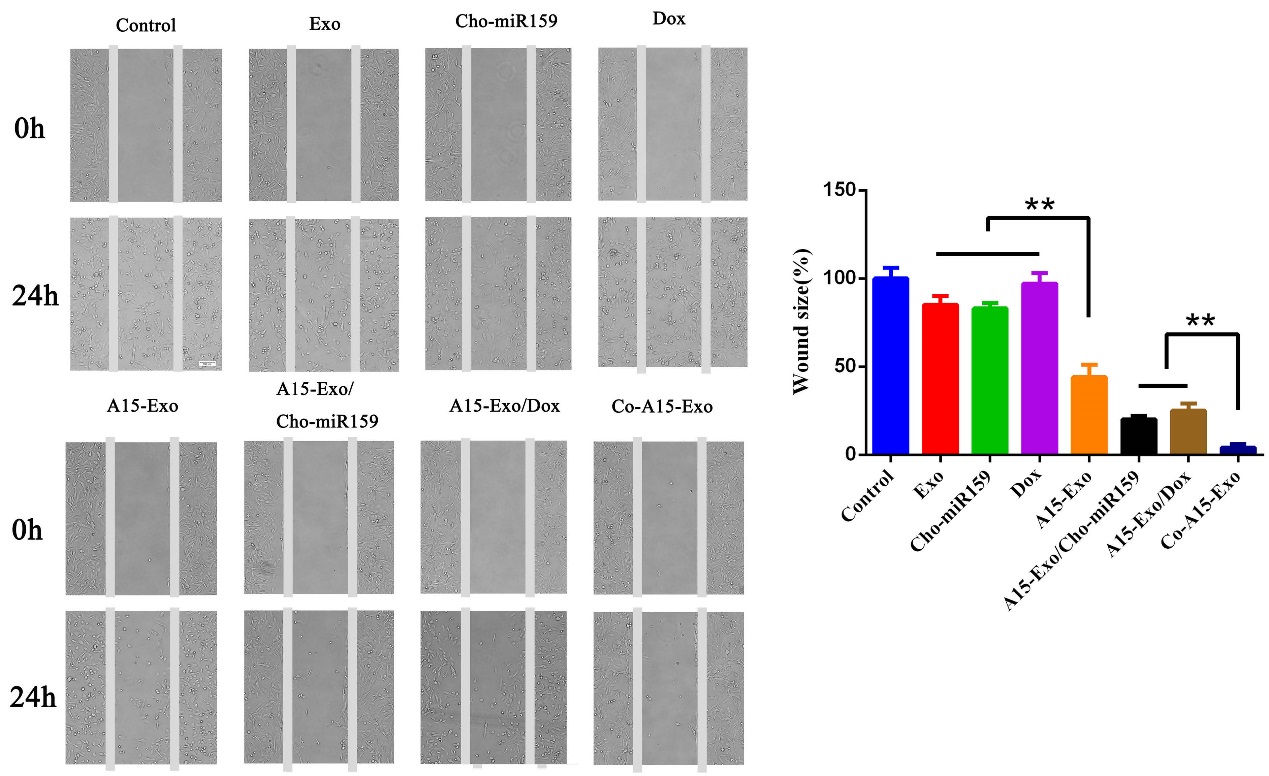
**

Figure S3 Wound healing assay of MDA-MB-231 cells incubated with Co-A15-Exo displayed arrested healing/closing of the scratch. (representative pictures from 3 repeated experiments) Scale bar: 100 μm.

**4. Transwell migration assay**

**
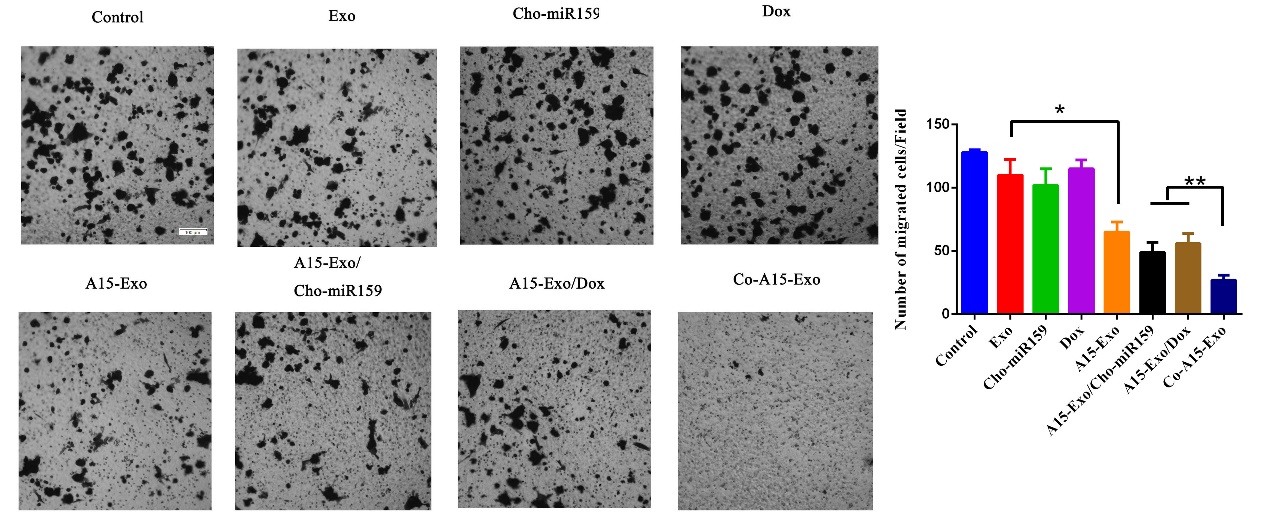
**

Figure S4 The migratory ability of MDA-MB-231 receiving different treatments was further confirmed by the transwell assay and quantitative analysis of the migrated cells. n = 3 per group. Scale bar, 100 μm.
